# Positive-Pressure Extubation Improves Early Oxygenation but Not Desaturation After Cesarean Delivery Under General Anesthesia: A Randomized Controlled Trial

**DOI:** 10.3390/healthcare14131991

**Published:** 2026-07-04

**Authors:** Nurdan Yılmaz, Yaşar Gökhan Gül

**Affiliations:** Department of Anesthesiology and Reanimation, Medipol Mega University Hospital, Istanbul Medipol University, Istanbul 34083, Türkiye; yasar.gul@medipol.edu.tr

**Keywords:** positive-pressure ventilation, oxygenation, postoperative hypoxemia, cesarean delivery, general anesthesia, desaturation

## Abstract

**Highlights:**

**What are the main findings?**

- Positive-pressure extubation (PPET) improved early postoperative oxygenation within the first 10 min after extubation compared to NPET.
- PPET did not significantly reduce the incidence of clinically relevant desaturation (SpO_2_ < 92%) within the first 60 min.

**What are the implications of the main findings?**

- PPET provided transient improvements in early postoperative oxygenation without demonstrating a significant benefit in the primary clinical outcome.
- Overall postoperative respiratory outcomes were comparable between the two techniques in this low-risk obstetric population.

**Abstract:**

**Background/Objectives**: Extubation technique, postoperative oxygenation, and hypoxemia are critical determinants of respiratory outcomes after general anesthesia. This study evaluated whether positive-pressure extubation improves early postoperative oxygenation and reduces desaturation in patients undergoing cesarean delivery. **Methods**: In this prospective randomized controlled trial, 120 participants were randomly assigned to positive-pressure or suction extubation. The primary outcome was postoperative desaturation (SpO_2_ < 92%) within 60 min after extubation. Secondary outcomes included early post-extubation oxygenation and hemodynamic parameters. Multivariable logistic regression was used to identify independent predictors of desaturation. The trial was registered at ClinicalTrials.gov (NCT07251686. **Results**: Postoperative desaturation occurred in 13.3% of patients in the positive-pressure group and 20% in the suction group (*p* = 0.327). Mean SpO_2_ values were significantly higher in the positive-pressure group at 0, 5, and 10 min after extubation (*p* < 0.01), but were similar at later time points. Extubation technique was not independently associated with desaturation (OR 0.69, 95% CI 0.24–1.98; *p* = 0.491). **Conclusions**: Positive-pressure extubation improves early postoperative oxygenation but does not reduce clinically relevant desaturation compared with suction extubation. Overall postoperative respiratory outcomes were comparable between the two techniques in this low-risk obstetric population.

## 1. Introduction

Extubation technique and postoperative oxygenation are critical determinants of respiratory outcomes after general anesthesia. A range of complications may occur during this phase, such as obstruction, aspiration, laryngospasm, cough, hypoxemia, apnea, and bronchospasm; approximately 30% of major anesthesia-related complications are reported to occur during extubation or recovery [1].

Difficult extubation is defined by the American Society of Anesthesiologists (ASA) as the inability to maintain airway patency or adequate ventilation after removal of an airway device in patients with a known or suspected difficult airway [2]. Asai et al. reported that complications occurred more frequently after extubation than after intubation, with 3.8% of patients experiencing airway obstruction immediately after extubation or in the recovery unit [3].

Clinical guidelines do not provide standardized recommendations for extubation procedures; however, two techniques have been described in the literature. First, the traditional extubation technique, known as the negative-pressure extubation technique (NPET), involves inserting a suction catheter alongside the endotracheal tube (ETT), deflating the cuff, and removing the ETT under continuous suction throughout the entire procedure. Second, the positive-pressure extubation technique (PPET) involves cuff deflation and application of positive pressure through the trachea during extubation. Theoretically, during PPET, airflow between the ETT and the larynx pushes accumulated subglottic secretions upward, allowing them to be removed from the oral cavity. A recent scoping review has summarized the available evidence comparing these techniques, reporting potential short-term benefits of positive-pressure strategies on oxygenation, although findings remain heterogeneous and population-specific data are limited [4,5].

It has been reported that NPET may promote atelectasis, which reduces lung volume (functional residual capacity) and oxygen stores, shortening the possible onset of arterial hemoglobin oxygen desaturation [5]. In pediatric patients, Guglielminotti et al. demonstrated that PPET reduces the incidence of arterial hemoglobin oxygen desaturation and delays its onset [6]. Several studies in adult populations have compared positive- and negative-pressure extubation techniques, reporting potential short-term benefits of positive-pressure approaches on oxygenation and airway protection [7]. However, results are heterogeneous, and evidence in obstetric patients remains limited.

Pregnant patients represent a unique population with physiological respiratory changes that increase the risk of rapid oxygen desaturation. Decreased functional residual capacity and increased oxygen consumption predispose parturients to hypoxemia even during brief periods of apnea, such as those occurring during extubation [8,9]. In addition, airway edema may further complicate airway management [10].

Although extubation techniques have been studied in non-obstetric populations, evidence in obstetric patients remains limited, and findings may not be directly generalizable. Therefore, evaluating extubation strategies in this population is clinically relevant.

This study aimed to evaluate whether PPET provides better protection against early postoperative oxygenation impairment compared to NPET.

## 2. Materials and Methods

This prospective randomized controlled trial was conducted after approval by the institutional ethics committee (Decision No: 1065, Date: 28 August 2025). Written informed consent was obtained from all participants. The study was registered at ClinicalTrials.gov (Identifier: NCT07251686) and conducted in accordance with the Declaration of Helsinki (2013 revision). The sample size was calculated to detect a clinically meaningful difference in postoperative desaturation between groups. Assuming a desaturation rate of 20% in the NPET group and 10% in the PPET group, with a two-sided α of 0.05 and 80% power, 54 patients were required per group. These assumptions were derived from previously published studies reporting postoperative respiratory complications and oxygen desaturation following tracheal extubation, as well as supported by institutional clinical observations [11]. To account for potential dropouts, a total of 120 patients were enrolled. Patients scheduled for elective cesarean sections at Medipol Mega University Hospital between September and November 2025 were allocated to either the NPET or the PPET group during the weaning phase. All patients underwent preoperative evaluation and provided written informed consent the day before surgery. On the day of surgery, before anesthesia induction, patients were assigned to groups using a prespecified randomization procedure. Block randomization was used to ensure equal distribution of 120 pregnant patients into two groups. Random allocation was performed using a computer-generated sequence. Allocation concealment was achieved using sequentially numbered, opaque-sealed envelopes opened by the attending anesthesiologist immediately before extubation. All patients received general anesthesia with intravenous propofol (2 mg/kg) and rocuronium (0.6 mg/kg), followed by orotracheal intubation and volume-controlled ventilation (tidal volume, 8 mL/kg; frequency, 12/min; Positive End-Expiratory Pressure (PEEP), 5; FiO_2_, 0.4). Inclusion criteria included adult patients aged >18 years undergoing elective cesarean section under general anesthesia. Exclusion criteria included participation in another study, refusal to participate, uncooperative behavior, emergency surgery, ASA score > 3, chronic respiratory failure, obstructive sleep apnea, or anticipated difficult airway. The randomization sequence was generated by an investigator who was not involved in patient recruitment, intraoperative management, or postoperative outcome assessment. Allocation concealment was achieved using sequentially numbered, opaque, sealed envelopes, which were opened by the attending anesthesiologist immediately before extubation.

Extubation was performed when patients were awake, responsive, and breathing spontaneously with adequate tidal volume (≥6 mL/kg) and stable hemodynamics following complete reversal of neuromuscular blockade. Neuromuscular recovery was assessed using clinical criteria, including sustained head lift, adequate spontaneous ventilation, and responsiveness to verbal commands. Immediately before extubation, all patients received 100% oxygen (FiO_2_ 1.0) for 3 min to maximize oxygen reserve. The oral cavity was suctioned prior to cuff deflation.

In the NPET group, closed suction was performed through the endotracheal tube without disconnecting the patient from the breathing circuit, and continuous negative pressure was applied during cuff deflation and tube removal.

In the PPET group, patients were placed on pressure support ventilation with PEEP set at 6 cm H_2_O and pressure support initially set at 12 cm H_2_O. If tidal volume exceeded 6 mL/kg, pressure support was gradually reduced by 2 cm H_2_O increments. Extubation was performed when pressure support reached 8 cm H_2_O, while maintaining positive airway pressure during cuff deflation.

Intraoperative analgesia was provided using intravenous opioids (e.g., fentanyl) according to standard clinical practice. Postoperative analgesia was administered using a standardized institutional protocol.

After extubation, supplemental oxygen was administered via face mask at a standardized flow rate of 4–6 L/min and adjusted if necessary to maintain peripheral oxygen saturation (SpO_2_) ≥ 95%. During the first hour following tracheal extubation, airway complications (cough, airway obstruction, laryngospasm, aspiration of gastric contents), blood pressure, and heart rate were recorded. Patients with SpO_2_ < 92% were evaluated for negative-pressure pulmonary edema by chest radiography and blood gas collection. Postoperative oxygen saturation measurements and outcome assessments were performed by an investigator who was blinded to group allocation.

Patients were also monitored for clinically evident postoperative pulmonary complications, including clinically suspected atelectasis, pneumonia, and negative-pressure pulmonary edema, during the postoperative hospital stay.

### Statistical Analysis

The normality of continuous variables was assessed using the Kolmogorov–Smirnov test and visual inspection of histograms. Continuous variables are presented as mean ± standard deviation (SD), and categorical variables are expressed as numbers and percentages.

Patients were divided into two groups according to extubation technique (NPET and PPET). Continuous variables were compared using the independent-samples *t*-test, and categorical variables were compared using the chi-square test or Fisher’s exact test as appropriate.

Desaturation-free recovery during the first 60 min after extubation was analyzed using the Kaplan–Meier method and compared between groups with the log-rank test.

To identify independent predictors of postoperative desaturation (defined as SpO_2_ < 92% within the first 60 min), univariate logistic regression analyses were first performed. Variables with clinical relevance and/or *p* < 0.10 in univariate analysis were entered into a multivariable logistic regression model. Odds ratios (ORs) with 95% confidence intervals (CIs) were calculated. Continuous variables were entered into the regression model without categorization. Multicollinearity was assessed using variance inflation factors (VIFs). Model calibration was evaluated using the Hosmer–Lemeshow goodness-of-fit test.

A two-sided *p* value < 0.05 was considered statistically significant. All statistical analyses were performed using IBM SPSS Statistics version 26 (IBM Corp., Armonk, NY, USA).

## 3. Results

Baseline demographic and perioperative characteristics of the study population are presented in Table 1.

The primary endpoint, postoperative desaturation (defined as SpO_2_ < 92% within the first 60 min after extubation), occurred in 12 patients (20%) in the NPET group and 8 patients (13.3%) in the PPET group (Table 1). The absolute risk reduction associated with PPET was 6.7% (95% CI, −20.1% to 6.9%), corresponding to a relative risk of 0.67 (95% CI, 0.29–1.51).

In univariate analysis, preeclampsia and BMI showed a trend toward association with postoperative desaturation. However, in multivariable logistic regression analysis adjusting for age, BMI, asthma, and preeclampsia, the extubation technique was not independently associated with postoperative desaturation (PPET vs. NPET: OR 0.69, 95% CI 0.24–1.98; *p* = 0.491). Preeclampsia demonstrated a borderline association (OR 5.05, 95% CI 0.88–29.01; *p* = 0.069), while BMI, asthma, and age were not significant predictors (Table 2).

Serial postoperative oxygen saturation, heart rate, systolic blood pressure, and diastolic blood pressure measurements at 0, 5, 10, 30, and 60 min are presented in Table 3. Immediately following extubation, mean SpO_2_ was significantly higher in the PPET group than in the NPET group (98.8 ± 0.4% vs. 98.1 ± 1.3%; *p* = 0.001). This improvement persisted at 5 and 10 min (*p* = 0.002 for both) but disappeared thereafter, as SpO_2_ values became comparable between the two groups at 30 and 60 min (*p* > 0.05).

Heart rate was significantly lower in the PPET group at all measured time points (*p* < 0.05). Systolic blood pressure did not significantly differ between groups at any time point (*p* > 0.05). Diastolic blood pressure was significantly higher in the PPET group at 10 and 60 min after extubation (*p* = 0.011 and *p* = 0.019).

**Figure 1:**
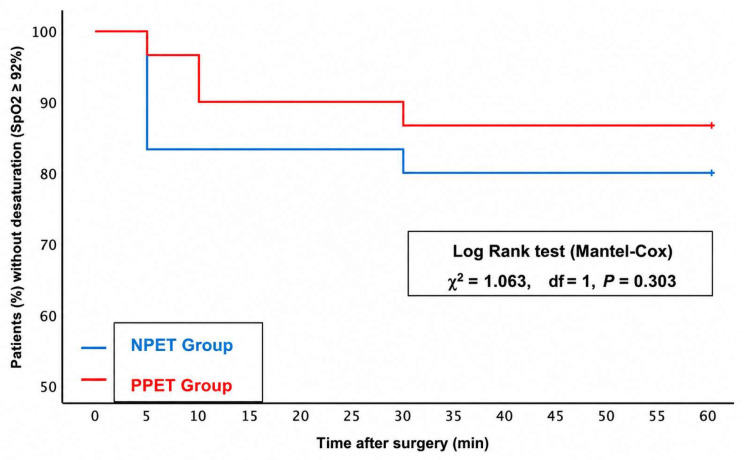
Kaplan–Meier analysis of desaturation-free survival during the first 60 min after extubation. NPET, negative-pressure extubation technique; PPET, positive-pressure extubation technique. “Kaplan–Meier curve showing desaturation-free survival within 60 min after extubation in patients undergoing cesarean delivery under general anesthesia. The x-axis represents time in minutes, and the y-axis represents probability of desaturation-free survival, comparing positive-pressure and suction extubation”.

Kaplan–Meier analysis demonstrated no statistically significant difference in desaturation-free survival between the NPET and PPET groups during the first 60 min after extubation (log-rank *χ*^2^ = 1.063, *df* = 1, *p* = 0.303; Figure 1). Although a slightly higher proportion of patients in the PPET group maintained SpO_2_ ≥ 92% in the early postoperative period, this advantage diminished over time. Overall, both extubation techniques were associated with high desaturation-free rates, with no significant difference between groups during the observation period.

No patient developed clinically evident postoperative pulmonary complications, including atelectasis, pneumonia, or negative-pressure pulmonary edema, and no patient required additional respiratory support during the observation period.

## 4. Discussion

In this randomized controlled trial, PPET was compared with NPET in patients undergoing cesarean section. Although no significant difference was found in the incidence of desaturation within 60 min postoperatively, which was our primary endpoint, the PPET was associated with better oxygenation, particularly during the first 10 min after extubation, and with lower heart rate and differences in diastolic blood pressure during follow-up. Impaired oxygenation is among the most frequently reported extubation-related complications and has been associated with reduced lung volume and diminished oxygen stores during endotracheal suctioning. Therefore, it has been the primary outcome measure in studies comparing conventional extubation techniques [5,6,12,13].

Although desaturation was less frequent in the PPET group, this difference did not reach statistical significance (*p* = 0.327).

Postoperative atelectasis is a significant risk factor for postoperative pulmonary complications [14,15]. Accordingly, various intraoperative mechanical ventilation strategies and extubation techniques have been explored to reduce atelectasis and postoperative pulmonary complications [16,17].

Studies evaluating extubation techniques have reported potential short-term benefits of positive-pressure approaches, although findings remain heterogeneous. Similar to our study, Andreu et al. reported a 13% reduction in the risk of major complications with positive pressure, although the clinical magnitude of this difference is unclear [18].

Although the PPET group demonstrated higher SpO_2_ values in the immediate post-extubation period, the absolute difference was small and limited to the first 10 min. This transient improvement did not translate into a significant reduction in clinically relevant outcomes such as desaturation. Therefore, the clinical significance of this finding appears limited. These findings indicate that PPET improves early oxygenation, particularly during the immediate postextubation period. Similar results have been reported by L’Hermite et al., who observed that positive-pressure extubation in adults improved oxygenation in the early postoperative period without increasing airway complications [5]. In a recent scoping review, Liu et al. also concluded that PPET offers short-term oxygenation benefits, although long-term pulmonary outcomes remain similar between techniques [4]. The distribution of obstetric and comorbid conditions, including asthma, preeclampsia, gestational diabetes mellitus, hypothyroidism, and twin pregnancies, did not differ significantly between the groups, and the duration of surgery and anesthesia was also similar (*p* > 0.05). The absence of a statistically significant difference in desaturation incidence may be attributed to the relatively low-risk obstetric population and short surgical duration. Cesarean populations are generally considered to have a lower baseline risk for postoperative pulmonary complications. Previous studies have similarly reported low desaturation rates in short procedures and nonobese patients, despite differences in extubation techniques [19].

Although postoperative desaturation occurred less frequently in the PPET group, the confidence intervals around the estimated treatment effect were relatively wide, indicating uncertainty regarding the magnitude of the true effect. Larger multicenter randomized trials are warranted to determine whether the observed reduction in desaturation translates into clinically meaningful benefits.

Heart rate was consistently and significantly lower in the PPET group at all measured time points (0, 5, 10, 30, and 60 min; *p* < 0.05). Systolic blood pressure did not significantly differ between groups (*p* > 0.05), but diastolic pressure was modestly but significantly higher in the PPET group at 10 and 60 min (*p* = 0.011 and *p* = 0.019). Similar findings were reported by Andreu et al., where hypertension and tachycardia were the most common complications and were observed more frequently in patients extubated with negative-pressure aspiration [18]. In this study, extubation may trigger adrenergic discharge, and severe coughing may contribute to transient hypertension [19,20,21]. A lower heart rate suggests that sympathetic stimulation related to airway manipulation has decreased or disappeared completely.

Shimada et al. emphasized that while PPET in the operating room is performed manually, intensive-care protocols often use ventilator-assisted mechanical PEEP. In our study, using ventilator-based positive pressure ensured standardization and avoided anesthesiologist variability, which may explain the consistent hemodynamic stability observed [7].

Multivariable logistic regression analysis did not identify extubation technique as an independent predictor of postoperative desaturation. Although preeclampsia showed a borderline association, BMI, asthma, and age were not significant predictors. In contrast to previous studies identifying obesity and respiratory comorbidities as risk factors for postoperative hypoxemia [22,23,24]. BMI was not associated with desaturation in our cohort, possibly due to the relatively homogeneous and low-risk obstetric population. Although preeclampsia demonstrated a borderline association with postoperative desaturation, only six patients in our cohort had this condition, precluding meaningful subgroup or sensitivity analyses. Future studies with larger cohorts should investigate whether the effects of positive-pressure extubation differ according to preeclampsia status, BMI, and other high-risk obstetric conditions. Although our study included a relatively low-risk obstetric population, the benefits of positive-pressure extubation may be greater in obese or morbidly obese parturients, who are at increased risk of perioperative hypoxemia because of reduced functional residual capacity and increased oxygen consumption [15,25]. Future studies should evaluate the effectiveness of this technique in these higher-risk populations.

PPET did not significantly reduce the incidence of postoperative desaturation compared with negative-pressure extubation in cesarean patients under general anesthesia. Although PPET was associated with transient improvements in early postoperative oxygenation, this effect did not translate into a reduction in desaturation. Both techniques appear to be safe and result in comparable clinical outcomes.

Although PPET improved oxygen saturation during the first 10 min after extubation, these modest improvements were limited to a secondary physiological outcome and did not translate into a significant reduction in the primary outcome of postoperative desaturation or other clinically relevant respiratory outcomes in this relatively low-risk population. Therefore, the clinical significance of these findings remains uncertain and should be confirmed in larger studies involving higher-risk obstetric populations.

In addition, all patients received standardized preoxygenation with 100% oxygen for 3 min before extubation in accordance with institutional practice. Although this approach ensured patient safety and standardized peri-extubation management, it may have attenuated differences in postoperative oxygenation between groups, potentially reducing the ability to detect a larger treatment effect. Therefore, the findings should be interpreted within the context of this standardized extubation protocol.

Accordingly, the findings of the present study should be considered generalizable primarily to low-risk women undergoing elective cesarean delivery under general anesthesia and should not be extrapolated to higher-risk obstetric populations without further evidence.

This study has several limitations. It was conducted in a relatively homogeneous, low-risk obstetric population undergoing elective cesarean delivery under general anesthesia, which may limit the generalizability of the findings to higher-risk or more diverse surgical populations. The primary outcome (SpO_2_ < 92%) was monitored only during the first 60 min postoperatively; therefore, delayed respiratory complications could not be assessed.

Another limitation is that quantitative neuromuscular monitoring was not used to confirm complete recovery before extubation. Although extubation was performed according to standardized clinical criteria following pharmacological reversal, objective neuromuscular monitoring could have further standardized extubation conditions and reduced potential variability between patients.

Additionally, atelectasis was not systematically evaluated using objective imaging modalities such as lung ultrasound or computed tomography, which limits direct assessment of the mechanistic impact of PPET on alveolar collapse. Future mechanistic studies incorporating lung ultrasound or other imaging techniques are warranted to determine whether the observed improvement in early postoperative oxygenation is associated with changes in postoperative lung aeration or atelectasis. Furthermore, although perioperative analgesia was administered according to standard clinical practice, variations in opioid dosing may have influenced postoperative respiratory parameters, including oxygen saturation.

Another limitation is that the sample size calculation was based on an expected treatment effect larger than the effect observed in this study. Consequently, the study may not have been sufficiently powered to detect the smaller between-group difference observed in the primary outcome. Therefore, these findings should be interpreted with caution and confirmed in larger multicenter randomized trials.

An additional limitation is that repeated postoperative physiological measurements were analyzed using separate comparisons at predefined time points rather than a repeated-measures statistical model. Although this approach allowed assessment of time-specific differences, it may have increased the risk of type I error due to multiple comparisons.

## 5. Conclusions

PPET offered a short-term improvement in postoperative oxygenation without significantly reducing clinically relevant desaturation in this low-risk obstetric population. Although the observed improvements in oxygen saturation were statistically significant, they were modest and limited to the immediate post-extubation period. Larger multicenter randomized trials in higher-risk obstetric populations are needed to determine whether these physiological improvements translate into clinically meaningful respiratory benefits.

## Figures and Tables

**Table 1:** Comparison of baseline characteristics and desaturation incidence between groups.

	NPET Group (n = 60, 50%)	PPET Group (n = 60, 50%)	Overall Population (N = 120)
Age, years	34.7 ± 5.5	32.2 ± 4.8	33.4 ± 5.3
Height, cm	163.1 ± 5.4	160.8 ± 5.8	161.9 ± 5.7
Weight, kg	88.5 ± 12.2	83.8 ± 9.5	86.1 ± 11.1
BMI, kg/m^2^	33.2 ± 4.2	32.3 ± 3.4	
ASA physical status, *n* (%)			
II	53 (88.3)	52 (86.7)	105 (87.5)
III	7 (11.7)	8 (13.3)	15 (12.5)
Asthma, *n* (%)	7 (11.7)	2 (3.3)	9 (7.5)
Preeclampsia, *n* (%)	5 (8.3)	1 (1.7)	6 (5)
Twin births, *n* (%)	3 (5)	2 (3.3)	5 (4.2)
Thalassemia minor, *n* (%)	2 (3.3)	3 (5)	5 (4.2)
Gestational DM, *n* (%)	5 (8.3)	4 (6.7)	9 (7.5)
Hypothyroidism, *n* (%)	5 (8.3)	7 (11.7)	12 (10)
Duration of surgery, min	49.1 ± 12.8	48.1 ± 10.2	48.6 ± 11.6
Duration of anaesthesia, min	56.2 ± 13.8	54.2 ± 10.1	55.2 ± 12.1
Desaturation within postop 60 min (SpO_2_ < 92%), *n* (%)	12 (20)	8 (13.3)	20 (16.7)

NPET, negative-pressure extubation technique; PPET, positive-pressure extubation technique; BMI, body mass index; DM, diabetes mellitus. Values are presented as mean ± SD or *n* (%).

**Table 2:** Univariate and Multivariable Logistic Regression Analysis of Predictors of Postoperative Desaturation.

	Unadjusted OR (95% CI)	*p* Value	Adjusted OR (95% CI)	*p* Value
PPET vs. NPET	0.62 (0.24–1.63)	0.334	0.69 (0.24–1.98)	0.491
BMI (kg/m^2^)	1.08 (0.99–1.18)	0.071	1.00 (0.90–1.10)	0.943
Asthma	3.10 (0.78–12.30)	0.109	0.98 (0.11–8.79)	0.983
Preeclampsia	4.85 (0.91–25.72)	0.064	5.05 (0.88–29.01)	0.069
Age (years)	1.02 (0.94–1.11)	0.602	0.99 (0.89–1.09)	0.835

OR, odds ratio; CI, confidence interval; NPET, negative-pressure extubation technique; PPET, positive-pressure extubation technique. Adjusted model includes extubation technique, age, BMI, asthma, and preeclampsia.

**Table 3:** Temporal changes in oxygenation and hemodynamic variables during the first 60 min.

	NPET Group (n = 60, 50%)	PPET Group (n = 60, 50%)	*p* Value
**SpO_2_, %**			
0 min	98.1 ± 1.3	98.8 ± 0.4	0.001
5th min	95.4 ± 3.7	97.1 ± 1.8	0.002
10th min	96.6 ± 1.6	97.2 ± 2.2	0.002
30th min	96.9 ± 2.1	97.2 ± 2.1	0.169
60th min	97.5 ± 1.3	97.9 ± 1.4	0.123
**Heart rate, bpm**			
0 min	102.7 ± 17.1	90.7 ± 12.6	<0.001
5th min	98.8 ± 18.8	86.7 ± 11.1	<0.001
10th min	93.8 ± 17.8	88.4 ± 7.5	0.037
30th min	92.2 ± 14.5	84.1 ± 11.8	0.001
60th min	88.8 ± 13.6	82.5 ± 10.2	0.007
**Systolic BP, mmHg**			
0 min	130.3 ± 18.2	125.7 ± 15.2	0.112
5th min	129.2 ± 12.9	125.9 ± 14.8	0.137
10th min	126.1 ± 14.2	125.9 ± 14.1	0.908
30th min	134.9 ± 19.4	129.1 ± 16.2	0.099
60th min	132.2 ± 17.3	130.7 ± 14.7	0.349
**Diastolic BP, mmHg**			
0 min	72.8 ± 9.7	73.8 ± 7.5	0.388
5th min	73.3 ± 10.5	71.8 ± 9.9	0.323
10th min	68.4 ± 8.8	72.2 ± 6.8	0.011
30th min	73.9 ± 10.4	73.6 ± 9.5	0.697
60th min	71.3 ± 10.1	75.7 ± 8.2	0.019

Values are presented as mean ± SD. NPET, negative-pressure extubation technique; PPET, positive-pressure extubation technique; BP, blood pressure; SpO_2_, peripheral oxygen saturation. Between-group comparisons at each time point were performed using independent-samples *t*-tests.

## Data Availability

The data presented in this study are available from the corresponding author upon reasonable request. The data are not publicly available due to ethical restrictions and patient confidentiality.

## Abbreviations

The following abbreviations are used in this manuscript:

PPET	Positive-pressure extubation technique
NPET	Negative-pressure extubation technique
ASA	American Society of Anesthesiologists
ETT	Endotracheal tube
PEEP	Positive End-Expiratory Pressure
PS	Pressure support
BMI	Body mass index
RM	Recruitment maneuver
